# Assessing catastrophic health expenditure and impoverishment in adult asthma care: a cross-sectional study of patients attending six public health clinics in Klang District, Malaysia

**DOI:** 10.1186/s12913-024-10731-8

**Published:** 2024-03-12

**Authors:** Norita Hussein, Chiu Wan Ng, Rizawati Ramli, Su May Liew, Nik Sherina Hanafi, Ping Yein Lee, Ai Theng Cheong, Sazlina Shariff Ghazali, Hilary Pinnock, Andrew Stoddart, Jürgen Schwarze, Ee Ming Khoo

**Affiliations:** 1 Department of Primary Care Medicine, Faculty of Medicine, Universiti Malaya, Kuala Lumpur, 50603 Malaysia; 2 Department of Social and Preventive Medicine, Faculty of Medicine, Universiti Malaya, Kuala Lumpur, 50603 Malaysia; 3 UM eHealth Unit, Faculty of Medicine, Universiti Malaya, Kuala Lumpur, 50603 Malaysia; 4 Department of Family Medicine, Faculty of Medicine and Health Sciences, Universiti Putra Malaysia, Serdang, 43400 Malaysia; 5 Edinburgh Clinical Trials Unit, Usher Institute, The University of Edinburgh, Edinburgh, UK; 6 NIHR Global Health Research Unit on Respiratory Health (RESPIRE), Usher Institute, Usher Institute, The University of Edinburgh, Edinburgh, UK; 7 Child Life and Health, Centre for Inflammation Research, The University of Edinburgh, Edinburgh, EH8 9AG UK

**Keywords:** Asthma, Catastrophic expenditure, Impoverishment, Financial risk protection

## Abstract

**Background:**

In Malaysia, asthma is a common chronic respiratory illness. Poor asthma control may increase out-of-pocket payment for asthma care, leading to financial hardships Malaysia provides Universal Health Coverage for the population with low user fees in the public health system to reduce financial hardship. We aimed to determine out-of-pocket expenditure on outpatient care for adult patients with asthma visiting government-funded public health clinics. We examined the catastrophic impact and medical impoverishment of these expenses on patients and households in Klang District, Malaysia.

**Methods:**

This is a cross-sectional face-to-face questionnaire survey carried out in six government-funded public health clinics in Klang District, Malaysia. We collected demographic, socio-economic profile, and outpatient asthma-related out-of-pocket payments from 1003 adult patients between July 2019 and January 2020. Incidence of catastrophic health expenditure was estimated as the proportion of patients whose monthly out-of-pocket payments exceeded 10% of their monthly household income. Incidence of poverty was calculated as the proportion of patients whose monthly household income fell below the poverty line stratified for the population of the Klang District. The incidence of medical impoverishment was estimated by the change in the incidence of poverty after out-of-pocket payments were deducted from household income. Predictors of catastrophic health expenditure were determined using multivariate regression analysis.

**Results:**

We found the majority (80%) of the public health clinic attendees were from low-income groups, with 41.6% of households living below the poverty line. About two-thirds of the attendees reported personal savings as the main source of health payment. The cost of transportation and complementary-alternative medicine for asthma were the main costs incurred. The incidences of catastrophic expenditure and impoverishment were 1.69% and 0.34% respectively. The only significant predictor of catastrophic health expenditure was household income. Patients in the higher income quintiles (Q2, Q3, Q4) had lower odds of catastrophic risk than the lowest quintile (Q1). Age, gender, ethnicity, and poor asthma control were not significant predictors.

**Conclusion:**

The public health system in Malaysia provides financial risk protection for adult patients with asthma. Although patients benefited from the heavily subsidised public health services, this study highlighted those in the lowest income quintile still experienced financial catastrophe and impoverishment, and the risk of financial catastrophe was significantly greater in this group. It is crucial to ensure health equity and protect patients of low socio-economic groups from financial hardship.

## Background

Asthma is a chronic respiratory illness with a global prevalence of 4.3% [1]. Asthma costs have increased globally, driven by the costs of emergency care and medications [2]. In Malaysia, the prevalence of asthma among adults is approximately 6.4% [3]. The Malaysian National Health and Morbidity Survey reported that every year, 20% of adults with asthma visited the emergency department for acute exacerbations [4]. A recent local study conducted in six public health clinics in Malaysia reported that 66% of clinic attendees still had poorly controlled asthma [5]. Most patients in this study reported regular clinic follow-ups and those with poor control were associated with frequent emergency visits [5]. Although asthma is a treatable condition, the high prevalence of suboptimal asthma control and frequent clinic visits has a substantial impact both on the use of public health care resources and individual health expenditure [3] as out-of-pocket payments may impose considerable financial burden on patients and families. An earlier study carried out at a tertiary centre in Malaysia reported that the mean monthly direct cost to individuals of asthma-related care was USD23 [6]. Another local study reported the cost of asthma-related care was mainly on medications and hospital admissions [7]. These studies were conducted in hospital settings. There is limited information on the direct cost of asthma on patients encountered at the primary care level.

Suboptimal asthma control potentially increases the need for urgent health care that patients have to pay for out-of-pocket. The World Health Organization (WHO) defines ‘out-of-pocket payments’ as direct payments made by households for health care [8]. High out-of-pocket payments are associated with catastrophic and impoverished spending and can potentially lead to financial hardship, especially among the poor [8]. Some low-income countries have poor financial health systems in healthcare or do not provide adequate financial protection, resulting in a high risk of catastrophic health expenditures [9, 10]. The Malaysian national healthcare system provides financial risk protection through universal access to a comprehensive package of government-guaranteed healthcare services and charging low user fees in the public health system [11, 12]. Malaysia has a dual health sector with public and private health systems [13]. The public health system, which comprises the public health clinics, is government-funded with a fee per clinic visit capped at Malaysia Ringgit MYR1 (USD 0.24) that covers consultation, basic investigations, and medications [14]. Government employees and pensioners, school-going children, and people aged 60 years and above are eligible to receive free health services in public health clinics [14]. All other groups, including the poor, are required to pay the MYR1 fee. In instances where medications are not available in public health clinics, patients (including those eligible for free health services) are required to buy prescribed medications from private pharmacies. The private health system comprises private hospitals or clinics that operate on a fee-for-service payment system in which patients pay out-of-pocket themselves or through their private health insurance purchased individually or through an employer-sponsored health scheme (6). Each individual is free to choose the public or private health services that they wish to use. In addition, some individuals choose complementary-alternative-medicine (CAM) for asthma treatment [15, 16], which is not subsidised by the government. These co-payments, albeit small in amount, and despite the financial risk protection provided for all patients could result in catastrophic expenses and impoverishment for vulnerable households especially in people with chronic and/or relapsing conditions. The equity in financial protection could also differ for different levels of socio-economic groups [12]. We therefore aimed to estimate the incidence of catastrophic health expenditure and medical impoverishment among adult patients with asthma in primary care settings and to examine factors associated with the risk of financial catastrophe.

## Methods

### Design, setting and participants

We undertook a cross-sectional questionnaire survey as part of the baseline data collection of patients recruited to an asthma cohort from six public health clinics in the Klang District, Selangor state, Malaysia [5]. The clinics were selected because they are part of the government-funded national healthcare provision where the consultation and treatment fees are capped at affordable levels. These clinics cater for 80% of the population in the district, the majority of whom are in the low and middle-income groups [12]. The prevalence of asthma in this area was reported to be 5.9% in 2011 [17]. The sample size of the cohort was calculated to enable the assessment of an improvement initiative using estimates from a meta-review of supported asthma self-management [18]. We recruited 1280 patients aged 5 years and above who had physician-diagnosed asthma and/or received asthma treatment in the previous year, allowing for an estimated dropout rate of 40% [19]. For this paper, we only analysed data for adults (≥ 18 years old).

### Data collection

We recruited patients during their scheduled or unscheduled visits to the clinics between July 2019 - January 2020 and used a questionnaire administered face-to-face [5] to collect baseline data. The questionnaire was developed based on a literature review and views from expert panels in primary care respiratory medicine [5]. The questionnaire consists of sociodemographic characteristics, healthcare use and related payments, current asthma status, comorbidities, and asthma treatment. It was given to six Family Medicine Specialists, two respiratory physicians, and five medical officers for face and content validity and several revisions were made to make items clearer and easier to comprehend [5]. All patients provided written informed consent. Data collected included patients’ demographic and socio-economic profile: age, gender, ethnicity, education level, working status, occupation, number of households, personal and household monthly income, main source of health payment, monthly out-of-pocket payment for direct costs of asthma-related care in outpatient clinics and asthma control using Global Initiative for Asthma (GINA) assessment of asthma control [20]. Patients self-reported their expenses on asthma-related costs in the one month preceding recruitment to the study. All costs were collected in Malaysia Ringgit (MYR) and converted to USD for reporting (currency exchange rate 4.203 for MYR to USD in 2019 [21]).

### Operational terminologies

- Direct costs of asthma-related care in outpatient clinics are out-of-pocket payments incurred for registration and consultation, medications, investigations, and any devices, and costs for transportation [22].
- Household income is the overall income earned by household members aged 18 years and above. Household income in Malaysia is classified into B40, M40, and T20, which refers to the Bottom 40%, Middle 40%, and Top 20% of the household income groups. B40, M40 and T20 refers to a household income of < MYR4850 (< USD1174.33), MYR4850–10,959 (USD1174.33–2653.51) and > MYR10959 (> USD2653.51) respectively [12].
- Incidence of poverty is the proportion of patients whose monthly household income falls below the poverty line. Malaysia has developed poverty lines for urban and rural populations in different states of the federation. This study used the poverty line of the urban population, MYR2022 (USD481.08), for the state of Selangor [12]. A household is considered poor if its total monthly income falls below the poverty line.
- Catastrophic health expenditure is the proportion of patients whose monthly out-of-pocket payments spent on direct medical costs exceeds 10% of their monthly household income. Catastrophic expenditure is health spending not covered by any public or private health scheme when the health cost spent is higher than the household income [12, 23].
- Medical impoverishment is calculated based on the poverty line of the state or country. It occurs when a household that is above the poverty line is pushed below the line after incurring household expenditures for healthcare. We used the Malaysian Poverty Line Income for 2019 stratified for urban and rural areas in the Selangor state [12, 23].
- Quintiles is a way of measuring income inequality. It ranks all households by income, from lowest to highest, and divides all households into five quintiles. This calculation measures the distribution of income among the five groups compared to the total. The first quintile (Q1) is the lowest fifth, the second quintile (Q2) is the next lowest, and so on [24].

### Data analysis

Statistical analysis was done using SPSS version 25.0. Descriptive statistics were used to analyse demographic and socio-economic data, and out-of-pocket payments for asthma-related care. All results, where appropriate, are presented as proportions (%), mean (standard deviation or standard error), and confidence intervals. The proportion of households that were below the poverty line and occurrences of catastrophic health expenditure and medical impoverishment across the different levels of quintile were calculated. Multivariate regression analysis was used to identify associations between demographic (age, gender and ethnicity), household income by quintiles, level of asthma control, and financial catastrophic risk. In this analysis, the Chinese ethnicity was grouped with ‘others’ as the numbers were small. We compared the outcome of catastrophic health expenditure risk for out-of-pocket payments, with and without the cost of transportation, to explore the impact of non-medical direct costs on health expenditure.

## Results

### Demographic and socio-economic profile

A total of 1,280 patients with asthma were recruited, 85% (*N* = 1092) were adults. All 1092 completed the face-to-face questionnaire. However, 8% of patients (*n* = 89) had missing data mainly in out-of-pocket payments (*n* = 22) and household incomes (*n* = 72), because they chose not to complete these questions. Hence, we analysed a total of 1003 patients. Table 1 shows patients’ demographic and socio-economic profile. The profile of the sub-group analysed (*N* = 1003) was similar to 1092 of the whole adult population. In the analysed group (*N* = 1003), the majority (81%) were from low-income households (B40 category). About half of the patients were working of which 88% worked in the private sector or were self-employed. More than half (65%) reported personal (patient’s and household’s) savings as their main source of health payment. The mean personal income was MYR1,317.90 (USD313.56) while the mean household income was MYR3,181.66 (USD756.99). Each household had an average of four people.

**Table 1:** Demographic and socio-economic profile of the overall adult population (*N* = 1092) and study population (*N* = 1003)

Profile		*N* = 1092	*N* = 1003
Age in years, mean (SD)		48.8 (15.5)	48.3 (15.1)
Gender, n (%)	Male Female	376 (34.4) 716 (65.6)	344 (34.3) 659 (65.7)
Ethnicity, n (%)	Malay Chinese Indian Others	590 (54.0) 111 (10.2) 374 (34.2) 17 (1.6)	551(55.0) 90 (8.9) 346 (34.5) 16 (1.6)
Level of education, n (%)	No formal Primary Secondary Tertiary	49 (4.5) 292 (26.7) 505 (46.3) 246 (22.4)	38 (3.8) 262 (26.1) 473 (47.1) 230 (22.9)
Occupation, n (%)	Not working Retired Working	418 (38.4) 132 (12.1) 542 (49.5)	374 (37.3) 113 (11.3) 516 (51.4)
Working sector, n (%)	Public Private Self-employed	65 (12.0) 398 (73.4) 79 (14.6)	60 (11.6) 380 (73.6) 76 (14.7)
*Household income category, n (%) (Classification in Malaysia)	B40 M40 T20	824 (75.5) 178 (16.3) 18 (1.7)	819 (80.9) 175 (17.3) 18 (1.8)
^#^Main source of health payment, n (%)	Personal savings Employment benefit Private insurance Others	667 (61.1) 165 (15.1) 56 (5.1) 132 (12.1)	654 (65.2) 159(15.8) 55 (5.5) 131 (13.1)
Household size mean (SD)		4 (1.85)	4.58 (1.63)
Personal monthly income, mean (SD) in MYR		1,258.00 (1,542.00)	1,317.90 (1,565.54)
Household monthly income, mean (SD) in MYR		3,168.00 (2,558.00)	3,181.66 (2,570.10)
^+^Asthma control Well-controlled Poorly controlled		372 (34.1) 720 (65.9)	338 (33.6) 665 (66.4)

*B40, M40 and T20 refers to household income of < MYR4850 (< USD1174.33), MYR4850–10,959 (USD1174.33–2653.51) and > MYR10959 (> USD2653.51) respectively ^17^

^#^ Main source of health payment: Personal savings refers to savings from personal and households; employment benefit refers to government, private and social security organisation (SOCSO) health scheme; others include free treatment, charity, non-government organisation

^+^GINA: Global Initiative for Asthma 2017

All currency in Table 1 in Malaysia Ringgit (MYR)

### Out-of-pocket payment for outpatient asthma-related care

Table 2 reports the direct costs of outpatient asthma care for the study population. The total mean monthly direct cost for asthma-related care was MYR22.33 (USD5.32). The mean monthly direct medical cost incurred by patients and households was MYR7.53 (USD1.79) and the mean direct non-medical cost, transportation cost was MYR6.37 (USD1.52). For patients who used CAM as a treatment for their asthma, the additional mean monthly cost spent was the highest at MYR8.43 (USD 2.01).

**Table 2:** Out-of-pocket payment for outpatient asthma-related care in the previous one month (*N* = 1003)

	Mean (MYR) (%)	SE (95% CI)
Total monthly direct cost	22.33	4.41 (13.67-31.00)
Outpatient clinic visit (s)	3.94 (17.65%)	0.63 (2.71–5.17)
Prescribed asthma medication(s)	1.68 (7.53%)	0.40 (0.90–2.47)
Over-the-counter asthma medication(s)	1.59 (7.12%)	0.31 (0.97–2.21)
Devices for asthma	0.32 (1.43%)	0.06 (5.87–6.88)
Transportation	6.37 (28.52%)	0.26 (5.87–6.88)
CAM for asthma	8.43 (37.75%)	4.28 (0.03–16.82)

All currency in Table 2 in Malaysia Ringgit (MYR)

### Catastrophic health expenditure and medical impoverishment

Table 3 shows the incidence of poverty among the patients and households. Even before considering healthcare payments, 41.6% were in the ‘poor’ category. Seventeen households (1.69%) reached the threshold for catastrophic healthcare payments none of whom were in the highest income quintile. The direct out-of-pocket payment for asthma-related care shifted three households from mid/high income (quintiles 3 and 4) below the poverty line, classifying them as meeting the threshold for medical impoverishment. Note that the estimation of impoverishment does not apply to households who were already below the poverty line. Excluding the cost of transportation (Table 4), overall, 14 households (1.40%) were catastrophic, however, patients who were in the lowest quintile (Q1) were the most affected.

**Table 3:** Catastrophic health expenditure and medical impoverishment in households by income quintile (including transportation costs)

	Out-of-pocket payment (including transportation costs)					
Quintiles of household income	Q1	Q2	Q3	Q4	Q5	Total
Complete data (N)	204	213	232	155	199	1,003
**Proportion meeting criteria for poverty at baseline**						
mean	100.0	100.0	0	0	0	41.5
SE						1.56
95% CI lower bound						38.5
95% CI upper bound						44.6
**Out-of-pocket payment as % of household income**						
mean	3.02	1.03	0.67	1.07	0.29	1.21
SE	0.80	0.23	0.09	0.65	0.04	0.20
95% CI lower bound	1.45	0.58	0.48	-0.20	0.22	0.82
95% CI upper bound	4.58	1.49	0.85	2.35	0.36	1.60
**Proportion meeting criteria for catastrophic health expenditure** (10% income threshold)						
mean	6.37	0.47	0.86	0.65	0.00	1.69
SE	1.71	0.47	0.61	0.65		0.41
95% CI lower bound	3.01	-0.45	-0.33	-0.62		0.89
95% CI upper bound	9.74	1.39	2.06	1.91		2.50
**Proportion meeting criteria for medical impoverishment**						
mean	*NA	*NA	0.43	0.65	0.00	0.34
SE			0.43	0.65		0.24
95% CI lower bound			-0.42	-0.62		-0.13
95% CI upper bound			1.28	1.91		0.81

*NA not applicable, patients in Q1 and Q2 were already below poverty line

**Table 4:** Catastrophic health expenditure and medical impoverishment in households by income quintile (excluding transportation costs)

	Out-of-pocket payment (excluding transportation costs)					
Quintiles of household income	Q1	Q2	Q3	Q4	Q5	Total
Complete data (N)	204	213	232	155	199	1,003
**Proportion meeting criteria for poverty at baseline**						
mean	100.0	100.0	0	0	0	41.5
SE						1.56
95% CI lower bound						38.5
95% CI upper bound						44.6
**Out-of-pocket payment as % of household income**						
mean	1.98	0.65	0.41	0.93	0.19	0.82
SE	0.74	0.23	0.09	0.65	0.03	0.19
95% CI lower bound	0.53	0.20	0.23	-0.34	0.12	0.45
95% CI upper bound	3.44	1.09	0.59	2.21	0.25	1.19
**Proportion meeting criteria for catastrophic health expenditure** (10% income threshold)						
mean	4.90	0.47	0.86	0.65	0.00	1.40
SE	1.52	0.47	0.61	0.65		0.37
95% CI lower bound	1.93	-0.45	-0.33	-0.62		0.67
95% CI upper bound	7.88	1.39	2.06	1.91		2.12
**Proportion meeting criteria for medical impoverishment**						
mean	*NA	*NA	0.43	0.65	0.00	0.34
SE			0.43	0.65		0.24
95% CI lower bound			-0.42	-0.62		-0.13
95% CI upper bound			1.28	1.91		0.81

*NA not applicable, patients in Q1 and Q2 were below poverty line

### Predictors of catastrophic health expenditure

Table 5 shows independent variables associated with catastrophic health expenditure.

The multivariate regression analysis showed that the only significant predictor of catastrophic health expenditure was household income (with or without including transportation costs). The higher income quintiles (Q2, Q3, Q4 & Q5) had lower odds of financial catastrophe than Q1; (OR 0.06; 95% CI 0.01–0.50), (OR 0.12; 95% CI 0.03–0.57) and (OR 0.04; 95% CI 0.00-0.31) respectively taking into account the confounders including transportation costs. Similarly, when adjusting for confounders excluding transportation costs. the higher income quintile (Q2, Q3, Q4 & Q5) had lower odds of financial catastrophe than Q1; (OR 0.08; 95% CI 0.01–0.62), (OR 0.14; 95% CI 0.03–0.68) and (OR 0.04; 95% CI 0.01–0.36) respectively. Age, gender, ethnicity, and poor asthma control were not significant predictors. When the cost of transportation was included for out-of-pocket payments, patients who were 65 years old and above had higher odds of catastrophic risk (OR 1.05; 95% CI 0.32–3.48) as compared to transportation costs were excluded (OR 0.57; 95% CI 0.13–2.47).

**Table 5:** Predictors of catastrophic health expenditure calculated with out-of-pocket payment (including and excluding the cost of transportation)

	Risk of catastrophic health expenditure (including the cost of transportation)		Risk of catastrophic health expenditure (excluding the cost of transportation)	
Variable	Odds ratio	95% CI	Odds ratio	95% CI
Age (years)				
18–65 65 and above (elderly)	1 1.05	0.32–3.48	1 0.57	0.13–2.47
Gender				
Female Male	1 0.50	0.15–1.68	1 0.74	0.21–2.54
HH income Quintiles				
Q1 Q2 Q3 Q4 and Q5	1 0.06 0.12 0.04	0.01-0.50-2 0.03–0.57 0.00-0.31	1 0.08 0.14 0.04	0.01–0.62 0.03–0.68 0.01–0.36
Ethnicity				
Malay Indian Others	1 0.58 1.01	0.19–1.76 0.19–5.20	1 0.60 1.53	0.17–2.04 0.28–8.24
Asthma control				
Poorly controlled Well-controlled	1 0.50	0.15–1.60	1 0.47	0.13–1.76

## Discussion

The World Health Organization (WHO) states that “direct payments or costs made to obtain health services should not expose people to financial hardship and should not threaten living standards” [25]. Protecting households against financial hardship has been conceptualised as ensuring financial protection [25]. Malaysia is committed to delivering the mission of the United Nations Sustainable Development Goals in achieving and sustaining universal health coverage [26] and this is illustrated by the findings of this study. It provides a free service for those working or retirees from the public sector, while others obtain treatment from public health clinics are heavily subsidised with just needing to pay a very minimal fee [26]. Asthma is a common chronic respiratory condition in Malaysia, with most patients seeking care at outpatient clinics either at the primary or secondary care level [27]. We looked at a sample of patients attending six government-funded public health clinics to determine if they had been provided with financial risk protection. Most of the patients in our study were from low-income groups (B40 category in Malaysia classification). Almost half (41.6%) of this population lived below the poverty line as defined locally. Half of these patients were working either in the private sector or self-employed. Personal savings were the main source of health payment in two-thirds of the studied population. The mean out-of-pocket payment was low at MYR22.34 (USD5.29) per month. The mean monthly out-of-pocket payments for CAM treatment were the highest at MYR8.43 (USD 1.99) among the direct costs. Although there is no or limited evidence to support its effectiveness, CAM is increasingly popular as a self-care medicine complementary to evidence-based asthma treatment [15]. A study in Malaysian health clinics revealed almost half of patients with asthma used CAM to complement their asthma medications [28]. CAM is regarded as a patient’s choice and is not subsidised in government-funded facilities, but interventions that acknowledge health beliefs on CAM could minimise out-of-pocket spending. The cost of transportation was the major direct non-medical cost incurred in a month. Previous studies have reported the bulk of catastrophic risk comes from the direct non-medical costs involving transportation and lodging [29–31]. Transportation is often cited as a major barrier to healthcare access and reducing the costs of transportation has been shown to help better access care, especially in low-income populations [32]. A strategy to improve transportation to health facilities could further assist in protecting those patients in lower income quintile from financial catastrophic risks. This study has demonstrated that catastrophic health expenditure and impoverishment due to asthma care were infrequent. We found 17 of the 1,003 patients in our study experienced catastrophic impact. After the incursion of direct health costs for asthma, three households (0.34%) were pushed into poverty. Although these findings could be considered low, there was already a considerably larger proportion of patients (41.6%) living below the poverty line. The proportion of household income used for out-of-pocket payment amongst those in the lower quintiles was greater compared to higher-income households, demonstrating a potential risk of financial hardship. This is evidenced by the regression analysis that showed patients who were in the higher income quintiles had lower odds of financial catastrophe. The findings of catastrophic health expenditure and impoverishment for asthma in this study were similar to earlier reports of the national household survey that stated a low incidence (2%) of catastrophic expenditure in Malaysia overall [33]. However, the level of poverty in our study was much lower at 0.34% compared to the World Bank report for Malaysia in 2009 which estimated it at 3.8% of the population [34]. Other countries in Asia, for example, Vietnam, have a relatively higher catastrophic expenditure and impoverishment for chronic non-communicable diseases overall, 14.6 and 7.6%, respectively [35]. Korea reported a catastrophic health expenditure of 7.6% for chronic disease [36]. However, these differences could be due to the socio-economic background of the studied population, differences in the provision of healthcare, and variations in the costs of resources (e.g., medications and investigations). Life expectancy in Malaysia has increased to about 75 years in recent years. Hence, the burden of non-communicable diseases is expected to rise [37] challenging the highly subsidised public health care system. Government policy will need to evolve to ensure that patients with low income and living in poverty are protected from financial catastrophic risks.

### Strengths and limitations of the study

Of the population in our study, 8% declined to provide financial information. These data may not have been missing at random as patients with very low incomes may have preferred not to divulge their poverty status. Nevertheless, this is representative of the population served by the public health clinics in this area. As this study recruited patients who visited the public health clinics for outpatient care, we may have missed some who were hospitalised and attending secondary care clinics – a group potentially more prone to catastrophic costs. Equally patients unable to afford transport may not receive care because they are unable to attend clinics even if the care is almost free. We may therefore have underestimated the incidence of catastrophe in the population. Another limitation is the accuracy in cost determination for types of out-of-pocket payment costs and the variation in the price of the medications bought from private pharmacies as data were self-reported. In addition, the indirect costs and cost of productivity loss were not captured in this analysis. Although this was not calculated, the findings of direct costs in this study reflect the impact of health spending on asthma care for poor patients.

## Conclusion

The financial risk protection provided by the Malaysian government lessens the financial burden for this population of people with asthma who are mostly in the lower socio-economic group. In general, patients and households in the Klang District have benefitted from the heavily subsidised public health services that are almost free. However, there was still a small proportion of patients in the lowest income quintile who still experienced financial catastrophe and impoverishment. Further steps are needed to ensure health equity so that patients in the low socio-economic group are protected from financial health risks at all times.

## Data Availability

The data that support the findings of this study are available from the corresponding author upon request.

## Abbreviations

MYR: Malaysian Ringgit
USD: United States Dollar
B40: Bottom 40%
M40: Middle 40%
T20: Top 20%
Q: Quintile
N/A: Not applicable
CAM: Complementary Alternative Medicine

## References

[1] To T, Stanojevic S, Moores G, Gershon AS, Bateman ED, Cruz AA, Boulet LP (2012). Global asthma prevalence in adults: findings from the cross-sectional world health survey. BMC Public Health.

[2] Bahadori K, Doyle-Waters MM, Marra C (2009). Economic burden of asthma: a systematic review. BMC Pulm Med.

[3] Chan YY, Teh CH, Lim KK et al. Lifestyle, chronic diseases and self-rated health among Malaysian adults: results from the 2011 National Health and Morbidity Survey (NHMS). BMC Public Health. 2015;15(1). 10.1186/s12889-015-2080-z PMC4527234 26246019

[4] NHMS. 2002. https://iku.moh.gov.my/images/IKU/Document/REPORT/2006/Asthma.pdf (accessed 12 October 2020).

[5] Hussein N, Liew SM, Hanafi NS, Lee PY, Cheong AT, Ghazali SS, Chinna K, Pang YK, Kassim A, Parker RA, Schwarze J, Sheikh A, Pinnock H, Khoo EM, RESPIRE Collaborators (2023). Asthma control and care among six public health clinic attenders in Malaysia: a cross-sectional study. Health Sci Rep.

[6] Chan PWK, Hussain S, Ghani NH, Debruyne JA, Liam CK (2002). The direct cost of treating bronchial asthma in a teaching hospital in Malaysia. Southeast Asian J Trop Med Public Health.

[7] Yong YV, Shafie AA (2018). How much does management of an asthma-related event cost in a Malaysian suburban hospital?. Value Health Reg Issues.

[8] https://www.who.int/data/gho/indicator-metadata-registry/imr-details/107 (accessed 12 May 2021).

[9] Rai S, Gautam S, Yadav GK, Niraula SR, Singh SB, Rai R, Poudel S, Sah RB (2022). Catastrophic health expenditure on chronic non-communicable diseases among elder population: a cross-sectional study from a sub-metropolitan city of Eastern Nepal. PLoS ONE.

[10] World health statistics 2017 (2017). Monitoring health for the SDGs, Sustainable Development Goals.

[11] Ng CW, Hairi NN, Ng CJ, Kamarulzaman A. Universal Health Coverage in Malaysia: Issues and Challenges (2014).

[12] Department of Statistics Malaysia. Household Income and Basic Amenities Survey Report 2019. 2020;3–57. Available from: https://www.dosm.gov.my/v1/index(accessed 9 May 2021).

[13] Hwong WY, Sivasampu S, Aisyah A, Shantha Kumar C, Goh PP, Hisham AN. National Healthcare Establishment & Workforce Statistics (primary care). National Clinical Research Centre; 2012.

[14] Fadzil F, Jaafar S, Ismail R (2020). 40 years of Alma Ata Malaysia: targeting equitable access through organisational and physical adaptations in the delivery of public sector primary care. Prim Health Care Res Dev.

[15] Koh WM, Abu Bakar AI, Hussein N (2021). Sociocultural influences on asthma self-management in a multicultural society: a qualitative study amongst Malaysian adults. Health Expect.

[16] Ramdzan SN, Pinnock H, Liew SM (2019). Perceptions of complementary/alternative medicine use and influence on evidence-based asthma medicine adherence in Malaysian children. npj Prim Care Respir Med.

[17] National Health and Morbidity Survey (NHMS). 2011. Available from: https://iku.moh.gov.my/images/IKU/Document/REPORT/NHMS2011 (accessed 12 Jan 2022).

[18] Pinnock H, Parke HL, Panagioti M (2017). Systematic meta-review of supported self-management for asthma: a healthcare perspective. BMC Med.

[19] National Health and Morbidity Survey (NHMS). 2019: Non-communicable diseases, healthcare demand, and health literacy—Key Findings. 2020. Available from: http://iku.gov.my/images/IKU/Document/REPORT/NHMS2019/NHMS2019Infographic.pdf (accessed 12 May 2021).

[20] Global Strategy for Asthma Management and Prevention Updated. 2020. Accessed May 12, 2021. www.ginasthma.org.

[21] World Development Indicators. The World Bank. 2019. Available from: https://data.worldbank.org/indicator/PA.NUS.FCRF?locations=MY.

[22] Merlis M. Family out-of-pocket spending for health services: a continuing source of financial insecurity. Commonw Fund. 2002;(509):4–15.

[23] O’Donnell O, van Doorslaer E, Wagstaff A, Lindelow M. 2008. Analyzing health equity using household survey data: A guide to techniques and their implementation: Catastrophic Payments in Health Care. World Bank Publications. 2008; 203–212. Available from: https://openknowledge.worldbank.org/handle/10986/6896.

[24] The World Bank. Available from: https://datahelpdesk.worldbank.org/knowledgebase/articles/1986160-what-are-quintiles.

[25] World Health Organization. Available from: https://www.who.int/health-topics/financial-protection.

[26] Sayuti M, Sukeri S (2022). Assessing progress towards sustainable development goal 3.8.2 and determinants of catastrophic health expenditures in Malaysia. PLoS ONE.

[27] Lim HM, Sivasampu S, Khoo EM, Mohamad Noh K (2017). Chasm in primary care provision in a universal health system: findings from a nationally representative survey of health facilities in Malaysia. PLoS ONE.

[28] Alshagga MA, Al-Dubai SA, Muhamad Faiq SS, Yusuf AA (2011). Use of complementary and alternative medicine among asthmatic patients in primary care clinics in Malaysia. Annals Thorac Med.

[29] Kieu TTM, Trinh HN, Pham HTK (2020). Et alDirect non-medical and indirect costs of diabetes and its associated complications in Vietnam: an estimation using national health insurance claims from a cross-sectional. surveyBMJ Open.

[30] Ibrahim N, Pozo-Martin F, Gilbert C. Direct non-medical costs double the total direct costs to patients undergoing cataract surgery in Zamfara state, Northern Nigeria: a case series. BMC Health Serv Res 2005; 163. 10.1186/s12913-015-0831-2 PMC4404068 25881013

[31] Shrime MG, Hamer M, Mukhopadhyay S (2017). Effect of removing the barrier of transportation costs on surgical utilisation in Guinea, Madagascar, and the Republic of CongoBMJ. Global Health.

[32] Syed ST, Gerber BS, Sharp LK (2013). Traveling towards disease: transportation barriers to health care access. J Community Health.

[33] Van Doorslaer E, O’Donnell O, Rannan-Eliya RP (2007). Catastrophic payments for health care in Asia. Health Econ.

[34] World Development Indicators.: Data by Country, Malaysia. The World Bank. Available from: https://databank.worldbank.org/source/world-development-indicators.

[35] Van Minh H, Xuan Tran B (2012). Assessing the household financial burden associated with the chronic non-communicable diseases in a rural district of Vietnam. Glob Health Action.

[36] Choi JW, Choi JW, Kim JH, Yoo KB, Park EC (2015). Association between chronic disease and catastrophic health expenditure in Korea. BMC Health Serv Res.

[37] Lo Ying Ru J, Allotey P. World Health Day 2018– Lessons from Malaysia on Universal Health Coverage. World Health Organisation. 2018. Available from: https://www.who.int/malaysia/news/commentaries/detail/world-health-day-lessons-from-malaysia-on-universal-health-coverage.

